# Genome Editing Technologies for Rice Improvement: Progress, Prospects, and Safety Concerns

**DOI:** 10.3389/fgeed.2020.00005

**Published:** 2020-06-04

**Authors:** Kashaf Zafar, Khalid E. M. Sedeek, Gundra Sivakrishna Rao, Muhammad Zuhaib Khan, Imran Amin, Radwa Kamel, Zahid Mukhtar, Mehak Zafar, Shahid Mansoor, Magdy M. Mahfouz

**Affiliations:** ^1^Agricultural Biotechnology Division, National Institute for Biotechnology and Genetic Engineering (NIBGE), Constituent College of Pakistan Institute of Engineering and Applied Sciences, Faisalabad, Pakistan; ^2^Department of Biotechnology, Balochistan University of Information Technology, Engineering and Management Sciences (BUITEMS), Quetta, Pakistan; ^3^Laboratory for Genome Engineering and Synthetic Biology, Division of Biological Sciences, 4700 King Abdullah University of Science and Technology, Thuwal, Saudi Arabia

**Keywords:** genome editing, CRISPR-Cas9, crop improvement, rice, CRISPR-Cas12a, base editors, safety concerns, transgene-free

## Abstract

Rice (*Oryza sativa*) is an important staple food crop worldwide; to meet the growing nutritional requirements of the increasing population in the face of climate change, qualitative and quantitative traits of rice need to be improved. Stress-tolerant crop varieties must be developed with stable or higher yields under stress conditions. Genome editing and speed breeding have improved the accuracy and pace of rice breeding. New breeding technologies including genome editing have been established in rice, expanding the potential for crop improvement. Recently, other genome editing techniques such as CRISPR-directed evolution, CRISPR-Cas12a, and base editors have also been used for efficient genome editing in rice. Since rice is an excellent model system for functional studies due to its small genome and close syntenic relationships with other cereal crops, new genome-editing technologies continue to be developed for use in rice. In this review, we focus on genome-editing tools for rice improvement to address current challenges and provide examples of genome editing in rice. We also shed light on expanding the scope of genome editing and systems for delivering homology-directed repair templates. Finally, we discuss safety concerns and methods for obtaining transgene-free crops.

## Introduction

**Figure 1:**
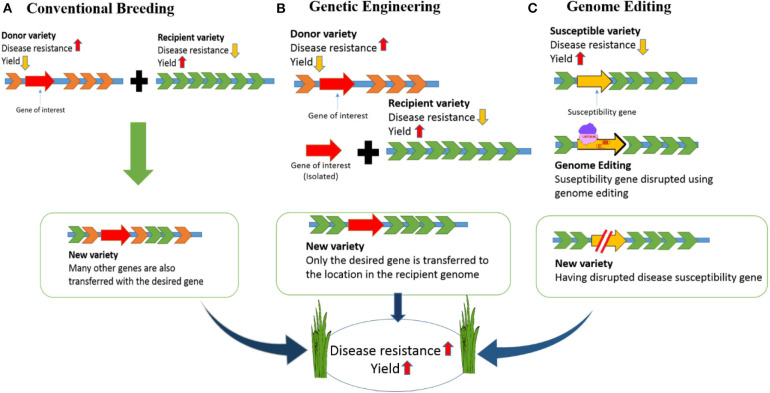
Developing disease-resistant rice: Comparison of conventional breeding, genetic engineering and genome editing. **(A)** In conventional breeding there will be cross of a donor variety having disease resistance and commercial variety having high yield but susceptible to disease. The new variety developed in this way will be disease resistant with high yield. However, undesired genes from the donor variety will be incorporated along with the desired gene. **(B)** For developing a disease-resistant variety through genetic engineering, we need to isolate the desired gene from the donor variety and introduce into the commercial variety. This will result in a GMO crop. **(C)** In genome editing the susceptible gene will be targeted and disrupted. So, the new variety created in this way will be disease resistant with high yield.

To address major issues including the growing population, environmental changes, and food scarcity, rice (*Oryza sativa*) varieties must be developed with higher yields and tolerance to environmental stress (Clarke and Zhang, 2013). Different strategies have been used to increase rice quality and yields. For example, although conventional breeding methods have improved, there have only been small increases in crop yields in recent years (Mann, 1999; Ansari et al., 2017). Genetic engineering has also been accomplished in rice but engineered crops have not reached end-users mainly due to public acceptance and political issues. Ultimately, genome engineering technologies offer expanded potential for crop improvement because they allow specific alterations of DNA sequences to be performed *in vivo* (Figure 1).

Over the past several years, CRISPR (Clustered Regularly Interspaced Short Palindromic Repeats) with CRISPR-associated protein Cas9 (CRISPR-Cas9) has transformed the field of genome engineering and emerges as novel system for genome editing. This technique is versatile and simple compared to other genome-editing tools such as zinc finger nucleases and transcriptional activator like effector nucleases (TALENs) (Cong et al., 2013; Miao et al., 2013; Ma X. et al., 2015). CRISPR-Cas9 is economical, easy to use, highly accurate and effective even when performing multiplex genome editing (Wang et al., 2017b). Multiplex genome editing is important because it allows multiple genes to be manipulated at several genomic locations. CRISPR-Cas9 targets specific genes and creates double-stranded breaks (DSBs) at the desired site. These DSBs are then repaired by the host's repair machinery, which performs homology-directed repair (HDR) or non-homologous end joining (NHEJ) to produce genomic alterations, gene knockouts and gene insertions. In NHEJ, the repair is performed by the host without the need for donor template, whereas in HDR, a donor template is required to repair the DSB. The frequency of HDR is lower than NHEJ, making the use of HDR in plants very challenging (Puchta, 2004).

CRISPR-Cas9 has multiple uses and is a rapidly growing area in the field of plant genome editing, especially in rice. Rice is an excellent model system for studying functional genomics due to its small genome size and close syntenic relationships with other cereal crops. Therefore, various genome-editing technologies viz., CRISPR-Cas9, CRISPR-Cas12a, and base editing continue to be developed for use in rice (Li et al., 2012; Feng et al., 2013). The current review focuses on different genome editing strategies for improving rice and their potential for addressing current challenges. We also focus on the safety concerns of rice genome editing and methods for obtaining transgene-free crops.

## Genome-Editing Technologies in Rice

In addition to the classic genome editing that requires the generation of DSB and harnesses the NHEJ or HDR pathway for the repair, new research continues to find innovative ways to alter the rice genome such as base editors and prime editing.

### CRISPR-Cas9

**Figure 2:**
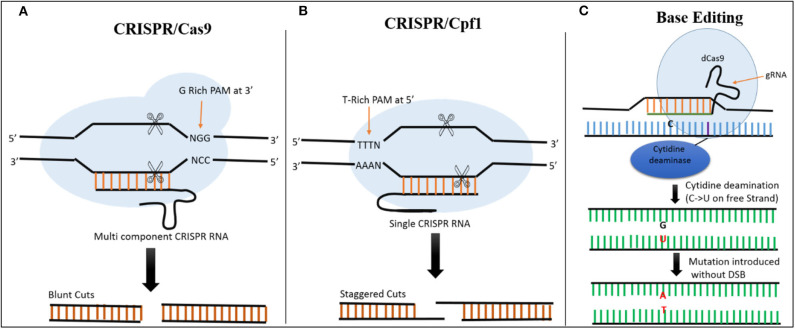
Comparison of CRISPR/Cas9, CRISPR/Cas12a, and Base Editing. **(A)** In CRISPR/Cas9 system, Cas9 is a multicomponent protein and recognizes a canonical G-rich PAM at the 3' end of the target site. CRISPR RNA and trans-activating CRISPR RNAs are required to recruit Cas9. The Cas9 creates a DSB resulting in blunt ends. **(B)** In the CRISPR/Cas12a System, Cas12a is a single-component protein which recognizes T-rich PAM at the 5' end of the target sequence. No trans-activating CRISPR RNA is required. The DSB results in staggered cuts. **(C)** In base editing cytidine deaminase fused with dCas9 is used to target the desired location. There is no DSB, C is converted directly into U on the free strand, and during mismatch repair a C → T substitution can be created when the modified strand is used as template.

In CRISPR-Cas9 systems, the base pairing of the single guide RNA (sgRNA) determines the specificity of Cas9-directed DNA cleavage. The protospacer adjacent motif (PAM) NGG, a nucleotide triplet, is required by Cas9 to create a DSB at the target site (Figure 2) (Cong et al., 2013). This system has been successfully exploited in plants. For example, as a proof-of-concept, (Feng et al., 2013) used the CRISPR-Cas9 system to target the *ROC5, SPP*, and *YSA* genes in rice and produced plants with an albino phenotype, indicating that this system can be successfully employed for targeted genome editing in rice.

Targeting the same locus at multiple sites or targeting multiple sites in different genes requires multiplex genome editing systems. In an effort to develop multiplex genome editing in rice, the tRNA processing system was used to generate multiple gRNAs from a single gene. In this system, tandemly arrayed tRNA–gRNA architecture is constructed [known as polycistronic tRNA–gRNA (PTG)] and each gRNA is attached to the target spacer sequence. The PTG primary transcript is processed and cleaved by the endogenous RNase P and RNase Z. This releasing multiple gRNAs with the desired sequence *in vivo* which directing Cas9 to edit multiple locations in the genome (Xie et al., 2015). This system was highly effective in rice, with up to 100% efficiency (Xie et al., 2015). This tRNA processing system was also used to deliver the donor repair template (DRT) for HDR. A chimeric sgRNA was used for dual purposes. This type of sgRNA included specific sequences to generate DSBs and a DRT to direct HDR. The chimeric sgRNA was synthesized as gRNA—gRNA Scaffold—pre-tRNA—DRT—Terminator. This system was used to generate herbicide resistance in rice and could potentially be used for targeted genome editing to improve other rice traits (Butt et al., 2017).

### Expanding the Target Sites for Editing

CRISPR-Cas9 is generally limited to perform genome editing at sites with canonical NGG PAMs. Much effort has focused on overcoming this restriction. Numerous Cas9 orthologs have been developed with altered PAM specificities, such as *Staphylococcus aureus* Cas9 (SaCas9) and Cas9-VQR (D1135V/R1335Q/T1337R) (Kleinstiver et al., 2015; Hu et al., 2016). The CRISPR-SaCas9 toolset was recently re-optimized by introducing three key mutations and its activity analyzed in rice. The newly optimized system performed genome editing with a mutagenesis efficiency of up to 77% (Qin et al., 2019).

A VQR variant of Cas9 was developed in rice that cleaves sites containing the PAM NGA, but the editing efficiency was low. In an effort to improve the editing efficiency of the VQR variant, the structure of the sgRNA was modified and strong endogenous promoters were utilized. These modifications significantly increased the editing efficiency of the VQR variant. This improved CRISPR-Cas9-VQR system is an excellent tool for genome editing, even at NGA PAM sites (Hu et al., 2018). Wild-type *streptococcus pyogenes* Cas9 (SpCas9) is able to recognize the NAG motif as well, making this PAM suitable for genome editing in rice with relatively low off-target effects. The rice genes *RAD51A* and *DMC1* were selected for editing using NAG as the PAM, and a mutation frequency of up to 85% was obtained (Meng et al., 2018).

Other versions of Cas9 have also been tested in rice, including expanded PAM SpCas9 (xCas9) and Cas9 that can recognize relaxed NG PAMs (Cas9-NG) (Nishimasu et al., 2018; Zhong et al., 2019). These enzymes can recognize a diversity of PAMs, including NG and GAA. Although the xCas9 system was successfully used for genome editing in rice, its efficiency was relatively low (Endo M. et al., 2019; Wang et al., 2018). Cas9-NG recognizes several non-canonical PAM sites such as NAC, NTG, NTT, NCG in addition to NG and GAT, GAA, and CAA (Ren et al., 2019; Zhong et al., 2019). These studies have greatly increased the scope of genome editing in rice.

### Base Editing

Although CRISPR-Cas9 and CRISPR-Cas12a systems have been developed for use in rice, both of these tools require DNA breakage in the form of DSBs. These breaks are then repaired by HDR (for insertions or replacement) or NHEJ (to knock out a gene), resulting in modification at the targeted sites (Jinek et al., 2012; Zetsche et al., 2015). The use of base editors allows precise editing to be performed at the desired location without the need for DSBs or donor template, making it more efficient than the other techniques for producing desirable mutations at the target site (Hess et al., 2017; Yang et al., 2017). The fusion of Cas9 nickase and cytidine deaminase enzyme facilitates the alteration of cytidine (C) to uridine (U), allowing C → T or G → A substitutions to be created at desired locations in the genome (Figure 2) (Komor et al., 2016).

Base editing was performed in rice by Zong et al. (2017). The authors targeted *OsCDC48* (which regulates senescence and cell death) to determine whether this system could be used to create nucleotide substitutions in rice. The target regions were successfully substituted, with a mutation frequency of 43.48%. There were no off-target mutations and no indels in the target region (Zong et al., 2017). In another study, a C-to-T substitution was created in *OsALS* to confer herbicide resistance in rice. An alanine-to-valine substitution at position 96 provided the rice plants with resistance to the herbicide imazamox (Shimatani et al., 2017). Additional base editing systems include BE4 (Komor et al., 2017), Targeted-AID (Komor et al., 2017), and dCpf1-BE (Li X. et al., 2018). All of these base editors use Cas9 or Cas12a nickase fused with cytidine deaminases leading to specific C-to-T substitutions.

Other established base editors include adenine base editors, which were developed by fusing SpCas9 nickase with tRNA adenosine deaminase. Adenine base editors were successfully used to create A·T to G·C conversions at target sites in human cells (Gaudelli et al., 2017). To examine the efficiency of adenine base editors in rice, *OsSPL14* was targeted. This gene is important for creating ideal plant architecture. A point mutation in *OsSPL14* at the binding site for the microRNA OsmiR156 was created with adenine base editors and successfully disrupted the cleavage of *OsSPL14* transcripts, resulting in enhanced grain yield and ideal plant architecture in rice (Hua et al., 2018a). This system creates A^*^T to G^*^C substitutions at a frequency of up to 59.1% in regenerated rice plants. Multiple endogenous rice genes were targeted, including a gene for herbicide tolerance, and herbicide-tolerant rice plants were developed by introducing a gain-of-function point mutation. Adenine base editors expanded the toolset for precise genome editing in rice (Li C. et al., 2018).

A new base editing system was developed in rice that combines the use of cytosine and adenine base editors with engineered SaCas9 and SpCas9 variants. This method increases the scope of base editing, as a wide range of target sites can be edited in the rice genome with different efficiencies. Cytosine and adenine base editing were successfully performed simultaneously in rice, highlighting the value of these new base editors for rice improvement (Hua et al., 2018b). Zong et al. (2018) used this system to modify the editing efficiency of base editors by fusing human APOBEC3A with Cas9 nickase. This fusion protein, which efficiently converts cytidine to thymidine, was used to perform genome editing in rice with an increased editing window size (Zong et al., 2018). The editing window was increased from 5 nucleotides using rat APOBEC1-based BE3 to a 17-nucleotide sequence, providing another example of the successful use of fusion proteins for efficient genome editing in rice. There are many other recent examples where base editing toolkits have been improved to increase editing efficiency, such as the rice-codon optimized adenine base editor (ABE)-nCas9 tool (Hao et al., 2019; Negishi et al., 2019), SpCas9-NGv1 (Negishi et al., 2019), and ABE-P1S (Hua et al., 2020).

### Improving HDR-Mediated Gene Knock-in and Insertion

Efficient HDR is crucial for precise genome editing in rice. Although CRISPR-Cas9 has become a promising genome-editing tool in rice and other crops, NHEJ is still preferred over HDR for DSB repair. The reason for that, is the difficulty of delivering enough repair template and the short time stability of the template inside the cell. Due to these difficulties, random insertion/deletion (indels) are primarily created at precise locations (Cong et al., 2013; Feng et al., 2013; Ma X. et al., 2015; Gao et al., 2016).

It is quite challenging to deliver a donor repair template (DRT) into a plant for HDR-mediated repair of a DSB. Various efforts have been made to improve HDR-mediated genome editing in rice and many of these center around the template for repair. For example, a geminivirus-based donor template delivery system was established in rice, which achieved a 19.4% gene knock-in frequency (Wang et al., 2017a). RNA templates can also be used for transcript-templated HDR (Li et al., 2019a). However, primary transcripts are processed and transported to the cytosol, making them unavailable for HDR (Li et al., 2019a). Coupling CRISPR/Cas12a to CRISPR RNA flanked with ribozyme, and a DRT flanked with either of these, produces specific primary transcripts capable of self-processing and releasing crRNA and the DRT in the nucleus. Stable rice lines were recently developed in which the rice *ACETOLACTATE SYNTHASE* (*OsALS*) gene was replaced with a mutated version harboring two specific mutations (Li et al., 2019a). The ALS enzyme is targeted by different herbicides and mutations at selected positions in *OsALS* confer herbicide resistance. To improve HDR, another effort was recently made in which Cas9 was fused with *Agrobacterium* VirD2. The Cas9-VirD2 chimeric protein has the dual functions of creating DSBs using Cas9 and bringing the DRT closer to the site of the DSB using VirD2 relaxase. The close proximity of the DRT facilitates HDR at the target site. This newly developed Cas9-VirD2 system was used for the precise modification of the *OsALS* allele in order to develop herbicide-tolerant rice plants (Ali et al., 2020).

### Gene Knock-in via Prime Editing

In plants, homology-directed repair (HDR) application is limited by extremely low efficiency (Butt et al., 2017; Ali et al., 2020). Many attempts have been made to improve it with little success due to the difficulty of delivering enough concentration of the repair template at the cut site (Baltes et al., 2014; Gil-Humanes et al., 2017; Wang et al., 2017a; Ali et al., 2020). Recently, Anzalone et al. (2019) have presented a new elegant method called prime editing that shows a great potential of generating different types of precise editing in mammalian cells with high efficiency. This method uses two components system, (1) an RNA-programmable nickase (Cas9-H840A) fused to reverse transcriptase (RT), and (2) a prime editing guide RNA (pegRNA) that specifies the genomic target site and encodes the desired edit. Compared to the HDR method, prime editing does not require a donor repair template, in contrary, the desired edits are incorporated into the pegRNA sequence. The RT uses the pegRNA as a template to copy the desired edits into the genome (Anzalone et al., 2019; Lin et al., 2020). Recently, several attempts have been made to establish an efficient prime editing system in rice and achieved herbicide-tolerant varieties (Li H. et al., 2020; Lin et al., 2020; Xu et al., 2020). They successfully targeted nucleotide substitution of the rice endogenous acetolactate synthase (*ALS*) gene with frequencies of up to 26% (Xu et al., 2020) and 14.3% (Lin et al., 2020) and 5-enolpyruvylshikimate-3-phosphate synthase (*EPSPS*) gene with frequency 2.22% (Li H. et al., 2020). This system is pretty new and few studies show its promise of the precise targeted modification in plants, However, it needs further optimization of the components and conditions.

### CRISPR-Cas12a/Cpf1

CRISPR-Cas12a (previously CRISPR-Cpf1) is a genome editing system that includes Cas12a, a class 2 CRISPR effector. Cas12a is a single RNA-guided endonuclease that, unlike Cas9, facilitates vigorous DNA interference. Cas12a recognizes T-rich PAMs and does not require tracrRNA (Zetsche et al., 2015; Alok et al., 2020). The DSBs created by Cas12a are also different from those of Cas9, as it creates staggered cuts (shown in Figure 2). The identification of this mechanism expands the application of genome editing, as it enables the editing of AT-rich regions (Like untranslated and promoter regions). However, this possess a constraint, as other sites lacking TTTN motifs cannot be recognized and edited with this system, thus limiting its usage in plants. However, this constraint has been overcome by developing modified Cas12a to identify other PAMs (Gao et al., 2017). This system was successfully used to target two rice genes, *OsPDS* and *OsBEL*. The plants were stably transformed at high efficiency, generating heritable, specific mutations. Preferably, can be utilized for different types of editing in rice genome (Xu et al., 2017). The editing capability of CRISPR- Cas12a and multiplexing in rice was also demonstrated using *OsPDS* and *OsSBEIIb*, encoding a phytoene desaturase and starch branching enzyme IIb, respectively. Mutating the target sites in these genes led to a loss of function and plants with an albino phenotype, confirming the potential of CRISPR-Cas12a for rice improvement through genome editing (Miao et al., 2018). The efficiency of the Cas12a system in rice was recently investigated by knocking out OsEPFL9 gene, involved in regulating stomatal density in leaves. The OsEPFL9 knockout rice lines showed significant reduction in stomatal count and increased water use efficiency under stress conditions (Yin et al., 2019). The efficiency of CRISPR-Cas12a was further improved by analyzing the temperature sensitivity of Cas12a. This system was highly active at 28°C, leading to a 93% mutagenesis frequency in T_o_ rice lines (Malzahn et al., 2019).

### CRISPR-Directed Evolution

In directed evolution, genetic diversity is increased artificially, giving rise to protein variants that are screened and selected for improved fitness. Enhanced plant traits and crop improvement can be achieved by increasing genetic diversity using directed evolution. A CRISPR-Cas9-based platform was established for rice improvement using directed evolution. Several mutant variants of SF3B1 conferring resistance to splicing inhibitors have been generated using this platform as a proof-of-concept for this technique. The successful production of SF3B1 variants was confirmed using structural studies of the binding of splicing inhibitors to these variants. The molecular functions of many important biomolecules can be altered using the directed evolution approach. Directed evolution can also be used to engineer crop traits to improve plant performance and help plants better adapt to climate change (Butt et al., 2019). A novel saturated targeted endogenous mutagenesis editors (STEMEs) was used to direct the artificial evolution of herbicide-resistant rice. In which, two single-base editors (cytidine deaminase and adenosine deaminase) are fused to produce simultaneous double-base conversions (C → T and G → A) in the rice acetyl-coenzyme A carboxylase gene (*OsACC*) (Li C. et al., 2020).

## Potential Uses of Genome Editing for Rice Improvement

**Figure 3:**
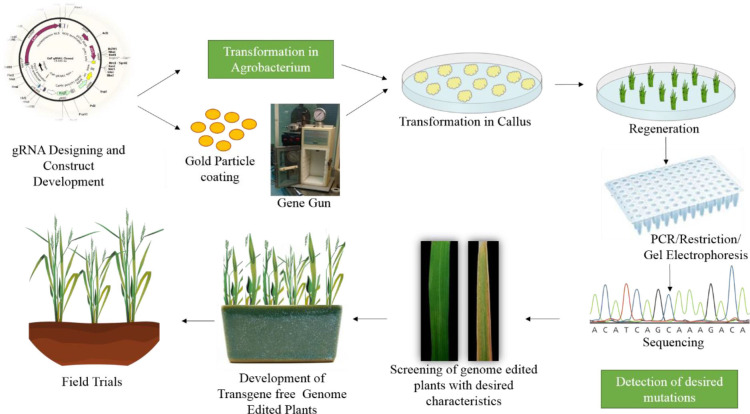
General strategy for improving rice through genome editing. The first step is gRNA design and construct development. After confirmation of the construct it is transferred either in *Agrobacterium* or coated on gold particles for biolistic bombardment. The construct is transformed to rice callus. The plants are regenerated from this callus. The regenerated plants are confirmed through PCR and restriction digestion, followed by sequencing. The plants are then screened for desired characteristics and moved to the greenhouse and then to field trials.

**Figure 4:**
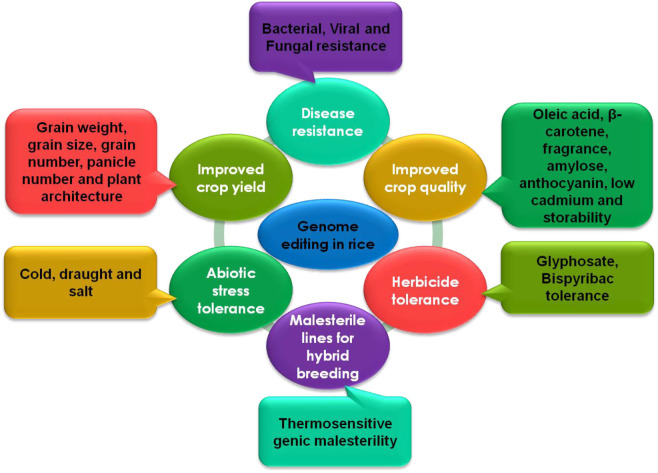
CRISPR-Cas mediated genome editing in rice for the improvement of different traits.

Many studies have demonstrated the successful use of genome editing to improve rice. The general strategy for rice improvement is shown in Figure 3. Below are some examples of the improvement of rice traits through genome editing (Figure 4, Table 1).

**Table 1:** Genes targeted by genome-editing systems in rice.

**Sr.#**	**Gene**	**Effect of gene on plant**	**Genome-editing system**	**References**
1	*ROC5, SPP, YSA*	Disruption results in albino phenotype	CRISPR-Cas9	Feng et al., 2013
2	*OsPDS* and *OsSBEIIb*	Encodes phytoene desaturase and starch branching enzyme	CRISPR-Cas12a	Li C. et al., 2018
*3*	*OsCDC48*	Regulates senescence and cell death	Base Editor C-to-T substitution	Zong et al., 2017
*4*	*OsALS*	Confers herbicide resistance	Base Editor and CRISPR-Cas9	Sun et al., 2016 Shimatani et al., 2017 Li et al., 2019a
*5*	*OsSPL14*	Gene for ideal plant architecture	Base Editor	Hua et al., 2018b
6	*Gn1a, GS3*	Grain yield	CRISPR-Cas9	Shen et al., 2018
7	Gn1a, DEP1	Grain yield	CRISPR-Cas9	Huang et al., 2018
8	*PYL1, PYL4, PYL6*	Control plant growth and stress responses.	CRISPR-Cas9	Miao et al., 2018
*9*	*OsFAD2-1*	Converts oleic acid into linoleic acid	CRISPR-Cas9	Abe et al., 2018
10	*Badh2*	Controls rice fragrance	CRISPR-Cas9	Shan et al., 2015 Shao et al., 2017
*11*	*SBEI, SBEIIb*	Control amylose contents	CRISPR-Cas9	Sun et al., 2017
12	*OsNramp5*	Metal transporter gene	CRISPR-Cas9	Tang et al., 2017
13	*LOXs*	Affect seed storability	TALEN-based genome editing	Ma L. et al., 2015
14	*TIFY1a, TIFY1b*.	Plant adaption to cold	CRISPR-Cas9	Huang et al., 2017
*15*	*SAPK2*	Functions in ABA-mediated seed dormancy	CRISPR-Cas9	Lou et al., 2017
16	*OsSWEET11 OsSWEET13 OsSWEET14*	Bacterial blight susceptibility genes	TALEN-based genome editing CRISPR-Cas9	Cai et al., 2017 Zhou et al., 2015 Jiang et al., 2013
17	*eIF4G*	Candidate rice tungro disease resistance gene	CRISPR-Cas9	Macovei et al., 2018
18	OsERF922	Responsible for rice blast resistance	CRISPR-Cas9	Wang et al., 2016
19	BBM1	Enables embryo formation from a fertilized egg	CRISPR-Cas9	Khanday et al., 2019
20	*REC8, PAIR, OSD1*, and *MTL*	For heterozygosity fixation and haploid induction	CRISPR-Cas9	Wang C. et al., 2019
21	*SF3B1*	Confers resistance to splicing inhibitors	CRISPR-directed evolution	Butt et al., 2019
22	OsEPFL9	Regulates stomatal density in leaves	CRISPR-Cas12a	Yin et al., 2019

### Improving Rice Yield

Grain yield is a complex yet crucial trait for rice improvement. Grain yield is controlled by many genes known as quantitative trait loci (QTLs). Various attempts have been made to improve grain yield using CRISPR-Cas9 (Sedeek et al., 2019). Four yield-related genes were successfully mutated in rice using CRISPR-Cas9, including *Gn1a* (gene for grain number), *DEP1* (*DENSE AND ERECT PANICLE1*; controls panicle architecture), *GS3* (regulates grain size), and *IPA1* (gene for plant architecture). Phenotypic variation was successfully generated due to the presence of edited genes, highlighting the efficiency of CRISPR-Cas9 for the targeted editing of genes regulating complex traits such as grain yield (Li M. et al., 2016). In a study, two important QTLs, *GRAIN SIZE3* (*GS3*) and *Grain number 1a* (*Gn1a*) were disrupted using CRISPR-Cas9 in five widely cultivated rice varieties. The same QTL was shown to have various (even contrasting) effects on grain yield in different genetic backgrounds (Shen et al., 2018). Similarly, three yield-related QTL genes (*OsGS3, OsGW2*, and *OsGn1a*) were edited simultaneously using multiplex genome editing. The resulting triple mutants showed a 30–68% increase in yield per panicle. This type of approach can be used for the rapid breeding of QTLs in elite crop varieties (Zhou et al., 2019).

In another study, superior alleles of yield-related genes were developed using CRISPR-Cas9. The authors were interested in determining whether alleles created by gene editing could have stronger effects than natural alleles. Two yield-regulating genes, Gn1a and DEP1, were edited to develop superior alleles in rice. The mutant alleles of Gn1a and DEP1 conferred higher yields than natural high-yield alleles, indicating that CRISPR-Cas9-based genome editing can be used to create superior alleles to improve current rice varieties (Huang et al., 2018).

Several phytohormones affect crop yields, including abscisic acid (ABA), which controls plant growth and stress responses. CRISPR-Cas9 was used to generate mutations in genes encoding the ABA receptors PYRABACTIN RESISTANCE 1-LIKE 1 (PYL1), PYL4, and PYL6, which created a rice line that produced up to 31% more grains than the original variety in field tests. This work highlights the potential of modifying ABA receptors to control growth and improve yields in rice (Miao et al., 2018).

## Improving Rice Quality and Nutrition

Although much effort has been expended to improve key agronomic traits such as yield, genome editing has also been used to improve the quality and nutritional value of rice. This includes altering the oil and starch compositions, manipulating the aroma, decreasing heavy metal accumulation, and biofortification efforts.

### Production of High Oleic/Low Linoleic Rice

Oleic acid is a valuable component of rice bran oil. Increasing the oleic acid content in rice bran oil would improve health and help prevent diseases. Oleic acid is converted to linoleic acid by the enzyme fatty acid desaturase 2 (FAD2) in plants. Rice contains three functional *FAD2* genes, with *OsFAD2-1* exhibiting the highest expression in seeds. By disrupting this gene, high oleic/low linoleic rice bran oil could be produced. Targeted mutagenesis of *OsFAD2-1* via CRISPR-Cas9 led to the creation of *OsFAD2-1* knockout rice plants with a 2-fold increase in oleic acid contents and no detectable linoleic acid, thereby improving the fatty acid composition of rice bran oil (Abe et al., 2018).

### Fragrant Rice

Fragrant rice is favored worldwide due to its high quality and excellent market value. Betaine aldehyde dehydrogenase (Badh2) is primarily responsible for fragrance in rice. The suppression of *Badh2* induces the biosynthesis of 2-acetyl-1-pyrroline (2AP). TALEN-based genome editing was used to disrupt *Badh2*, resulting in an increase in 2AP levels from 0.35 to 0.75 mg/kg. This amount of 2AP is almost equal to that found in the aromatic rice variety that was used as a positive control (Shan et al., 2015). CRISPR-Cas9 based genome editing of *Badh2* was also used to develop fragrant rice in the Zhonghua 11 (*indica* rice variety) background. The first exon of *Badh2* was edited to include an additional base (T), which led to an increase in 2AP contents. Such studies could help fast-track the breeding process of fragrant rice (Shao et al., 2017).

### Rice With Increased β-Carotene Accumulation

The biofortification of β-carotene is an important target for rice improvement. The *Osor* gene, an ortholog of the *Orange* (*Or*) gene in cauliflower, was targeted in rice using CRISPR-Cas9. *Or* is responsible for β-carotene accumulation in cauliflower curd. The directed modification of *Osor* via CRISPR-Cas9-based genome editing resulted in enhanced β-carotene accumulation in rice callus (Endo A. et al., 2019).

### Rice With Increased Amylose Content

Cereals with high amylose contents represent a good source of resistant starch (Regina et al., 2006). Foods with high resistant starch contents are nutritious and may reduce the risk for many diseases (Jiang et al., 2010). CRISPR-Cas9 technology was used for directed mutagenesis of *SBEI* and *SBEIIb* in rice; these genes control amylose content (Sun et al., 2017). Although there was no clear difference between *sbeI* edited plants and wild type, the *sbeII* edited rice plants showed considerably increased amylose and resistant starch contents, with increases up to 25 and 9.8%, respectively. This indicates the ability of CRISPR-Cas9 system to create high-amylose rice by editing *SBEIIb* gene.

### Low-Cadmium Rice

The presence of heavy metals in food can be harmful to human health. Eliminating heavy metals from soil would lessen their accumulation in rice grains. In addition, genome editing could be exploited to reduce the levels of toxic metals in food crops. Cadmium (Cd) is extremely toxic and dangerous for human health (Clemens et al., 2013). Rice is a major source of calorie intake, and its grains often contain excessive amounts of Cd. Therefore, Cd accumulation should be controlled in rice. Since it is challenging to develop low-Cd rice via conventional breeding, there is a need to develop new strategies for generating low-Cd rice lines. CRISPR-Cas9 has been used to develop low-Cd rice lines by knocking out the metal transporter gene *OsNramp5*. The *OsNramp5* mutants showed dramatically reduced Cd contents compared to the wild type. In field trials, the mutants contained <0.05 mg/kg Cd compared to 0.33 to 2.90 mg/kg in the control plants. The reduced Cd level was achieved without affecting yields (Tang et al., 2017).

In another study, CRISPR/Cas9-based editing was used to destroy *OsNRAMP5* in two *japonica* rice varieties. Three independent *OsNRAMP5* mutants were generated, and the effects of the mutations on Cd accumulation and plant growth were examined. Less Cd and Mn accumulated in the roots and shoots of the mutants compared to wild-type plants. However, in a paddy field experiment, many traits were affected in the mutants, including plant height, leading to lower grain yields. Therefore, the editing of *OsNRAMP5* should be approached with caution. Factors such as pH, soil conditions, and water must be considered, as they might influence the Mn level in the soil, ultimately affecting grain yield (Yang et al., 2019). These findings pave the way for investigating how genome editing could be used to minimize heavy metal contamination in crops.

### Red Rice

Red rice contains high levels of the health-promoting nutrients proanthocyanidins and anthocyanins. Two genes, *Rc* and *Rd*, are involved in determining the red coloration of rice grains. The *RcRd* genotype of wild rice species *Oryza rufipogon* produces red pericarp tissue, whereas many varieties of cultivated rice produce white grains due to a frame-shift deletion of 14-bp in the 7th exon of *Rc*. CRISPR-Cas9 was recently used to bring back the recessive *Rc* allele by reversing the frame-shift deletion into an in-frame mutation, thus converting white pericarp rice varieties into red ones. The red grains obtained from the mutants contained high levels of proanthocyanidins and anthocyanidins with no notable difference in agronomic traits between the wild type and mutants, indicating that reversing the functioning of Rc has no negative effect on major agronomic traits in rice. This approach could therefore be used for crop improvement (Zhu et al., 2019).

### Improving Storability

Seed longevity and quality are compromised during storage due to the deterioration of grains, causing severe economic losses for farmers. Lipoxygenases (LOXs) negatively regulate seed storability by catalyzing the dioxygenation of polyunsaturated fatty acids to form hydroperoxide. TALEN-based genome editing was successfully used to mutate *LOX3* in rice, resulting in LOX3 deficiency and improved seed storability (Ma L. et al., 2015).

## Herbicide and Abiotic Stress Tolerence

Abiotic stresses such as cold, drought, and salinity cause substantial yield reduction and pose challenges for crop production and feeding the growing world population. Engineering cultivars that tolerate such stresses is the most sustainable solution for this problem. The new advances in genome editing technologies enable fast and precise traits engineering for crop improvement.

### Herbicide Tolerance

Important herbicides such as chlorsulfuron and bispyribac-sodium (BS) are used to control a wide variety of grass and dicot weeds. These herbicides, which target the OsALS enzyme, are among the most widely used chemicals for weed control (Zhou et al., 2007). The targeting of OsALS by these herbicides results in the loss of enzymatic activity, hence blocking the biosynthesis of branched chain amino acids (valine, leucine, and isoleucine). TALEN-based genome editing was used to introduce point mutations into *OsALS* with an efficiency of 6.3% (Li T. et al., 2016). In a similar experiment, CRISPR-Cas9 was used to introduce point mutations into targeted regions of *OsALS* (Sun et al., 2016). The edited plants were screened phenotypically by spraying with the herbicide bispyribac sodium. The wild-type plants died 36 days after spraying with bispyribac sodium, whereas the edited plants were tolerant to the herbicide and grew normally. These experiments revealed the potential of genome editing for developing herbicide-tolerant rice plants. Herbicide resistance rice was also achieved by replacing the second exon of the rice endogenous gene, 5-enolpyruvylshikimate-3- phosphate synthase (*EPSPS*) with a new exon containing several nucleotide substitutions using CRISPR/Cas9. The modified EPSPS has two amino acid substitution (T102I and P106S) and confer resistance to the herbicide glyphosate (Li J. et al., 2016).

### Cold Tolerance

Rice is extremally sensitive to cold environment, especially at the seedling stage, therefore, Low temperature is considered as one of the major factors affecting rice growth and production. Engineering cold tolerance rice is important for plant survival and production. A recent study by Zeng et al. (2020) has employed CRISPR/Cas9 to improve the rice cold tolerance. They successfully knocked-out the endogenous *MYB30*, a cold tolerance gene, simultaneously with other two yield related genes. The triple mutant exhibited better cold tolerance and yield compared with the wild type plants.

### Drought Resistance

SNF 1-RELATED PROTEIN KINASE 2 (SnRK2), a member of the plant-specific protein kinase family, is an important regulator of osmotic stress responses and ABA signaling. The role of SAPK2 was comprehensively studies using CRISPR-Cas9. The edited mutants had non-functional SAPK2 and were insensitive to ABA, indicating that SAPK2 plays a key role in ABA-mediated seed dormancy. The *SAPK2* mutants were more sensitive to oxidative stress and drought than control plants, demonstrating the importance of SAPK2 for drought tolerance in rice. These findings suggest that *SAPK2* should be targeted for crop improvement in the future (Lou et al., 2017).

### Salt Tolerance

One of the major problems affecting rice production worldwide is salinity. The most environmentally friendly approach to controlling salinity is the cultivation of salt-tolerant rice varieties. CRISPR-Cas9 was used to target *OsRR22* in rice to improve salt tolerance (Farhat et al., 2019; Zhang et al., 2019). The mutant lines were examined for agronomic traits as well as salinity tolerance. The mutant lines showed enhanced salinity tolerance at the seedling stage compared to wild-type plants.

## Biotic Stress Resistance

Various bacterial, fungal, and viral pathogens attack rice plants, causing diseases that result in significant losses in crop yields and quality. Many strategies are used in the field to combat these diseases (Heinrichs and Muniappan, 2017). Genome editing technologies are also being applied to develop disease-resistant rice varieties.

### Bacterial Leaf Blight Resistance

Bacterial leaf blight (BLB) is a wide-ranging disease of rice caused by *Xanthomonas oryzae* pv*. oryzae* (*Xoo*), which poses a great threat to overall food security. Multiple strategies have been used to fight BLB, including conventional and molecular breeding to develop resistance against various *Xoo* strains (Pradhan et al., 2015). Pathogens evolve very rapidly, giving rise to new pathotypes to combat plant defense mechanisms, resulting in the need to develop advanced approaches for increasing disease resistance in rice. Xanthomonas contains type III effector proteins known as transcription activator-like effectors (TALEs), which target the *SWEET* gene family. SWEET proteins transport sugar into the apoplast (Cohn et al., 2014). *SWEET14* is the main target, as geographically distinct *Xoo* strains target this gene via different effectors such as AvrXa7, PthXo3, Tal5, and TalC. These effectors activate *OsSWEET14* to provide sugars for the pathogen. TALEN technology was used to disrupt an effector-binding element in the *OsSWEET14* promoter to develop plant resistance against bacterial blight (Li et al., 2012). The role of SWEET14 was studied comprehensively by editing different effector binding elements in the *SWEET14* promoter using TALEN-based genome editing, revealing that the effector TalC also targets an effector binding element in a gene other than *SWEET14*. However, *OsSWEET14* plays a major role in susceptibility to *Xoo*, as not only PthXo3 but other effectors such as TalC and AvrXa7 target *OsSWEET14* (Blanvillain-Baufumé et al., 2017).

CRISPR-Cas9 based genome editing was also used to develop *indica* rice lines with increased resistance to BLB by mutating *OsSWEET13* to prevent its interaction with pthXo2 (Zhou et al., 2015). TALEN-based editing was used to alter the effector-binding site of Tal7 in the promoter of Os09g29100, which decreased the severity of BLB (Cai et al., 2017). In another effort to develop BLB resistance, Cas9-gRNA constructs were designed to target the promoters of *OsSWEET14* and *OsSWEET11*, resulting in decreased BLB symptoms (Jiang et al., 2013). The same technology was then used to edit the effector binding sites of *SWEET* genes to create a new germplasm with broad-spectrum resistance against wide range of Xoo strains (Xu et al., 2019). Finally, in another study, elite mega varieties of rice (IR64 and Ciherang-Sub1) were edited using CRISPR-Cas9 to develop broad-spectrum resistance against BLB by introducing five mutations in the promoter region of three *SWEET* genes (Oliva et al., 2019). All of these efforts were fairly successful and could ultimately lead to the development of rice cultivars with resistance to Xoo.

### Rice Tungro Disease Resistance

Rice production in Asia is also severely affected by rice tungro disease. This disease is caused by the interaction of *Rice tungro bacilliform virus* (RTBV) and *Rice tungro spherical virus* (RTSV). Extensive studies to develop resistance to rice tungro disease have revealed *eIF4G* (*translation initiation factor 4 gamma*) as a candidate gene for RTSV resistance and that the Y1059V1060V1061 region of *eIF4G* interacts with RTSV (Lee et al., 2010). CRISPR-Cas9 genome editing was used to target *eIF4G* in an RTSV-susceptible rice variety in an effort to develop resistance against rice tungro disease. The mutations created in *eIF4G* were heritable, with no off-target effects. The edited plants showed resistance to RTSV, representing important materials for developing rice varieties with increased resistance to rice tungro disease (Macovei et al., 2018).

### Rice Blast Resistance

Rice blast, the most destructive disease of rice, is caused by the filamentous fungus Magnaporthe oryzae. This disease poses a serious threat to rice production worldwide (Liu et al., 2014). The most economical way to deal with rice blast is to develop rice varieties with resistance to this disease (Miah et al., 2013). CRISPR-Cas9 was used to target OsERF922, a gene responsible for rice blast resistance in rice. The targeted mutagenesis was successful, and a 42% mutation frequency was observed. The number of blast lesions was considerably reduced in the edited lines compared to the control plants, highlighting the value of CRISPR-Cas9 for improving rice blast resistance (Wang et al., 2016).

## Other Traits

### Male-Sterile Lines for Hybrid Rice Production

Disease-free male sterile lines have great potential for use in hybrid rice breeding. Using CRISPR-Cas9, mutations were introduced into male sterility-related genes *TMS5, Xa13*, and *Pi21*. The transgene-free mutants acquired in the T_1_ generation showed characteristics of thermosensitive genic male sterility and improved resistance to rice blast and bacterial blight. This approach provides a way to convert breeding materials into thermosensitive genic male sterile lines though gene editing, ultimately leading to accelerated breeding using male sterile lines (Li et al., 2019b).

### Reducing Seed Dormancy

Genome editing was also used to reduce seed dormancy in rice. The *viviparous-1* (*OsVP1*) gene, encoding a transcription factor involved in regulating of seed development and ABA signaling, was knocked out in rice using CRISPR-Cas9. The edited lines showed increased expression of ABA signaling genes, suggesting that genome editing could be effective for reducing seed dormancy in rice (Jung et al., 2019).

### Genome Editing to Develop Asexual Reproduction in Rice

Breeders often develop elite plant varieties with better yields, but these phenotypes are usually lost during genetic segregation. Various attempts have been made to preserve these phenotypes. Plants can sometimes form an embryo from a fertilized egg due the expression of “Baby Boom” (*BBM1*) in sperm cells. The expression of this gene in fertilized cells is due to a contribution from the male. Genome editing was used to remove the plant's ability to go through meiosis. Due to this disruption, the plant produced egg cells via mitosis, indicating that the full set of chromosomes was derived from the mother (Khanday et al., 2019). Subsequently, when the cells expressed BBM1, these plants developed egg cells able to generate embryos, which will then grow into clonal seeds. These plants could be reproduced clonally through seed propagation (Khanday et al., 2019). The strategy was also employed for clonal reproduction of F_1_ rice hybrids. Four genes, *REC8, PAIR, OSD1*, and *MTL* were edited using CRISPR-Cas9 for heterozygosity fixation and haploid induction. The plants produced in this way were able to proliferate clonally through seeds. This strategy can be exploited for the self-propagation of elite rice varieties developed by breeding (Wang C. et al., 2019).

## Transgene-Free Edited Rice and Safety Concerns

For genome editing, the Cas9 and the sgRNA that provides target specificity are generally expressed from transgenes that are integrated into the host genome. However, to maintain the stable phenotypes of edited plants, the CRISPR-Cas9 construct that is inserted in the genome must be removed, as the presence of CRISPR-Cas9 creates problems when trying to differentiate previously generated mutations from newly generated mutations. The chances of off-target effects also increase if CRISPR-Cas9 is present in the genome. Furthermore, genome-edited crops should be transgene free if regulatory approval is needed for commercial applications. Therefore, after modifying the genome, various methods are used to remove CRISPR-Cas9 components. This step is also required for the social acceptance of genome editing in agriculture. Here, we review some of the methods that have been developed to avoid off-target effects and make edited crops transgene free.

### Conventional Approaches for Removing the Transgene

Transgene removal has been performed using molecular methods such as the FLP/FRT, Cre/loxP, and piggyBac transposon systems. Other conventional approaches have also been used, including segregation. The content of the parental line can be maintained by backcrossing or selfing. Many studies have reported the development of Cas9-free lines after genome editing through segregation (Xu et al., 2015; Gao et al., 2016; Pyott et al., 2016; Nekrasov et al., 2017; Zaidi et al., 2018).

### Editing in Germ Line Cells

Germ line cells with the desired mutations can be edited in order to develop transgene-free plants. The CRISPR construct can be delivered to germ line cells using viral vectors, thereby creating the desired mutations in these cells. The seeds from these germ line-edited plants transfer the targeted mutations to the next generation. This system can be used to develop germ line-edited plants, but it is less efficient for crop plants (Ali et al., 2015; Zaidi and Mansoor, 2017).

### Fluorescence-Based Selection

An innovative strategy was used to develop Cas9-free genome-edited *Arabidopsis thaliana* plants. A cassette was added to the CRISPR-Cas9 vector that facilitates the expression of the mCherry gene. This cassette provides a visual screen to isolate plants free of Cas9. The seeds produced from the T_1_ generation segregated into two groups, including one with strong fluorescence and the other with no fluorescence. Since both the CRISPR-Cas9 and mCherry cassette were present on the same plasmid, the group with no fluorescence contained Cas9-free plants. This strategy made it easy to distinguish between seeds with and without the *Cas9* transgene. By exploiting this strategy, the workload for identifying transgene-free plants was reduced by 75% (Gao et al., 2016). A fluorescence-dependent module for monitoring the transgene was subsequently included in the genome editing toolbox using a modular cloning system. This approach has been tested in plants such as tomato (*Solanum lycopersicum*), rice, and Arabidopsis. The dry seeds allowed efficient visualization of DsRED fluorescence, which was used as a marker to identify the transgene, allowing transgene-free dry seeds to be selected. The genetic manipulation of specific targets in homozygous mutants was detected in the first generation of DsRED-free CRISPR-Cas9 null segregants. Using this approach, transgene-free homozygous plants were rapidly selected in a single generation after transformation (Aliaga-Franco et al., 2019).

### CRISPR Machinery With an RNA Interference Element

RNA interference (RNAi) has been used in plants to reduce the presence of undesirable elements and to develop resistance against viral diseases (Kamthan et al., 2015). RNAi works by inhibiting gene expression or neutralizing targeted mRNA molecules. Functional RNAi hairpins have a dominant nature and can be identified in T_0_ plants. An RNAi expression element was incorporated into a CRISPR-Cas9 construct to target a gene encoding P450, an enzyme responsible for herbicide resistance. This strategy provided herbicide-based separation of transgene-free genome-edited plants. The identification and separation of transgene-free plants through this approach is phenotype based and does not require PCR (Lu et al., 2017).

### CRISPR-Cas9 Ribonucleoproteins

Ribonucleoproteins (RNPs) are complexes of sgRNA and purified Cas9 protein that can be used to develop transgene-free genome-edited plants. The RNP is transformed into the plant by transfection or using a gene gun. This type of strategy for generating transgene-free plants was first reported by Woo (Woo et al., 2015). This method circumvents the need for transgene integration, because Cas9 performs its function without being integrated into the genome. The complex is then degraded in the cell. This type of genome editing is highly suitable for developing transgene-free genome-edited plants for commercial applications. The strategy was recently exploited to develop transgene-free edited rice resistant to blast fungus (Foster et al., 2018).

A genome-editing system was developed in which Cas9-gRNA RNPs were incorporated into plant zygotes. Cas9-gRNA RNPs were also directly delivered into rice zygotes generated by *in vitro*-generated outlying gametes. The zygotes were cultured in the absence of selection agents and converted to mature plants, resulting in regenerated rice plants with targeted mutations in 14–64% of plants. This efficient, effective plant genome manipulation technique could be used for the improvement of rice and other crop species (Toda et al., 2019).

### Transgene-Free Genome Editing by Transiently Expressing CRISPR-Cas9

Another genome-editing method for use in plants that are regenerated from callus is transient expression of CRISPR-Cas9, which can be introduced as RNA or DNA. This system, which is based on transient expression, is highly effective and precise for generating transgene-free plants. This method has been used in many crop plants (Zhang et al., 2016; Hamada et al., 2018) and can be used for transgene-free genome editing in rice.

### Suicide Transgene Method

Although several methods are effective for generating transgene-free plants, they are degraded soon, leading little time for performing targeted mutation. Therefore, another system was developed that vigorously and automatically eradicates CRISPR-Cas9-containing plants but provides sufficient time for the CRISPR system to perform the desired genome editing. A pair of suicide transgenes was employed to efficiently kill pollen and T_0_ plants containing a CRISPR-Cas9 construct. This strategy was successfully used in rice to generate targeted mutations and isolate transgene-free plants. This technique greatly reduces the time and labor needed to detect transgene-free plants containing mutations produced by CRISPR-Cas9 (He et al., 2018).

## Conclusions and Future Perspectives

Recently developed genome editing tools hold great potential for crop improvement because they enable precise modification at target sites. The main benefit of genome editing technologies is that protocols have been developed to eliminate the transgene, resulting in no difference between crops developed through genome editing and conventional breeding. Scientists are continuously trying to improve genome-editing systems by discovering new proteins or improving existing proteins in these systems. Recently, Cas9 was circularly permuted into protease-activated ProCas9s. This ProCas9s can sense protease activity in a cell, which is usually present during viral infection, and respond to it. This improved system will enable safer, more efficient genome modification (Oakes et al., 2019). New technologies have been also developed, such as CRISPR-Cas12a and base editing, which allow modifications to be made at the desired location in the rice genome. CRISPR-Cas9 and CRISPR-Cas12a can be used in combination to edit multiple locations in the rice genome with different PAM sequences. Multiplexing can also be performed using base editors by editing multiple genes responsible for different agronomic traits. Due to these advancements, multiple genes can be edited in a single experiment (Hua et al., 2018b). CRISPR-Cas12a was recently shown to target single-stranded DNA (ssDNA) as well as dsDNA. Cas12a can completely degrade ssDNA molecules, thus increasing the scope of genome editing to target ssDNAs (Chen et al., 2018).

Cas9 and Cas12a have been used for DNA manipulation, but the recently discovered Cas13a also targets ssRNA molecules. This enzyme was used to develop resistance against viruses in plants. As ssRNA viruses are a major class of viruses in plants, this system could be highly important for crop improvement (Aman et al., 2018). This system has also been used to visualize RNA molecules. Moreover, DNA and RNA manipulation can be combined to achieve complex changes in plants (Wolter and Puchta, 2018). Cas14, another new promising tool, is one-third the size of the Cas9 protein and can target ssDNA molecules very efficiently without a PAM restriction (Harrington et al., 2018). In the future, perhaps these systems could be used to manipulate any sequence in rice. However, plants should be developed by exploiting transgene-free genome editing protocols; these plants could possibly be regarded as non-GMO to increase the social acceptance of this newly emerging technology. We predict that genome-editing technology will result in a second green revolution to guarantee food security to meet the demands of an ever-increasing population.

To date, epigenetic genome modifications have not been reported in rice. Using dCas9 fused with methyltransferases, methylation levels in mammals can be manipulated and transferred to the next generation without any transgene integration (Liu et al., 2016). However, this type of system has yet to be developed for rice and other crop plants. Despite many improvements, the requirement for a specific PAM still poses a restriction to genome editing. Many strategies have been used to overcome this restriction, including modifying Cas9 to recognize different PAM sequences (Hu et al., 2018; Meng et al., 2018). The variant xCas9 3.7 was developed to expand the scope of genome editing in rice (Wang et al., 2018). A new version of Cas9 was also engineered (SpCas9-NGv1) that was able to recognize NG PAMs in rice (Oakes et al., 2019). Many other such variants do not work effectively in plants, highlighting the need to develop more Cas9 variants to recognize a wide range of PAMs. The limitation posed by PAM specificity can also be bypassed using the newly discovered Cas14a, as this system does not require any PAM, but it can only target ssDNA (Harrington et al., 2018).

**Figure 5:**
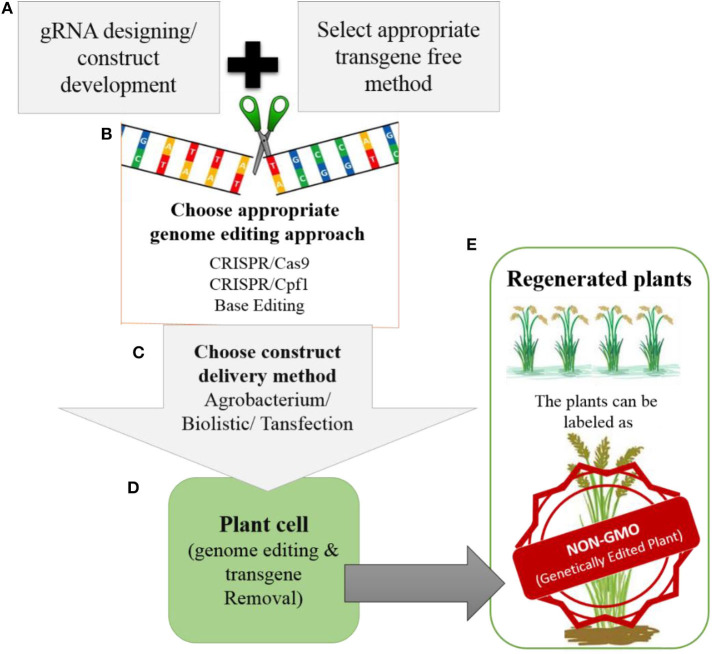
Development of transgene-free genetically edited rice plants for commercialization **(A)**. The gRNA will be designed for targeted modification and during construct development appropriate transgene-free method will be chosen (e.g., transient expression of Cas9, RNP or suicide transgene method etc.) (**B)**. For genome editing, the appropriate genome editing tool will be chosen depending upon the feasibility and type of modification that needs to be introduced. **(C)**. The developed construct can be delivered to plant by *Agrobacterium*, biolistic or transfection. **(D)**. Once the construct is delivered into the plant. It will perform its function by modifying the target site in the rice genome. After modification the transgene will be removed from the plants. **(E)**. The regenerated plants will be screened for targeted modification without transgene. The plants developed in this way will be with desired traits without any transgene integration in the genome, so can be labeled as NON-GMO genetically edited plants by regulatory authorities.

Most improvements in crops have involved targeted editing, which requires the repair of DSBs through HDR and the delivery of the donor template. Therefore, advancements are needed in methods for delivering donor templates and improving the efficiency of the HDR pathway. Truly successful genome editing for crop improvement requires the cultivation of edited crops in the field. However, most of the genome-edited crops reported to date have not yet reached the field due to biosafety and regulatory issues. Whether genome-edited plants should be considered non-GMOs is still a matter of debate. However, there is support for the notion that these plants could be labeled as non-GMO genetically edited plants (Figure 5), because technically there is no difference between plants produced through genome editing vs. conventional breeding once transgene-free protocols have been established.

## Author Contributions

KZ, GR, and KS prepared the figures and table. KZ, KS, GR, and MM edited and finalized the manuscript. All authors wrote the manuscript.

## Conflict of Interest

The authors declare that the research was conducted in the absence of any commercial or financial relationships that could be construed as a potential conflict of interest.
